# Line-Tension Controlled Mechanism for Influenza Fusion

**DOI:** 10.1371/journal.pone.0038302

**Published:** 2012-06-28

**Authors:** Herre Jelger Risselada, Giovanni Marelli, Marc Fuhrmans, Yuliya G. Smirnova, Helmut Grubmüller, Siewert Jan Marrink, Marcus Müller

**Affiliations:** 1 Theoretical Molecular Biophysics Group, Max-Planck-Institute for Biophysical Chemistry, Göttingen, Germany; 2 Institut für Theoretische Physik, Georg-August-Universität, Göttingen, Germany; 3 Groningen Biomolecular Sciences and Biotechnology Institute, Nijenborgh, AG Groningen, The Netherlands; German Primate Center, Germany

## Abstract

Our molecular simulations reveal that wild-type influenza fusion peptides are able to stabilize a highly fusogenic pre-fusion structure, i.e. a peptide bundle formed by four or more trans-membrane arranged fusion peptides. We rationalize that the lipid rim around such bundle has a non-vanishing rim energy (line-tension), which is essential to (i) stabilize the initial contact point between the fusing bilayers, i.e. the stalk, and (ii) drive its subsequent evolution. Such line-tension controlled fusion event does not proceed along the hypothesized standard stalk-hemifusion pathway. In modeled influenza fusion, single point mutations in the influenza fusion peptide either completely inhibit fusion (mutants G1V and W14A) or, intriguingly, specifically arrest fusion at a hemifusion state (mutant G1S). Our simulations demonstrate that, within a line-tension controlled fusion mechanism, these known point mutations either completely inhibit fusion by impairing the peptide’s ability to stabilize the required peptide bundle (G1V and W14A) or stabilize a persistent bundle that leads to a kinetically trapped hemifusion state (G1S). In addition, our results further suggest that the recently discovered leaky fusion mutant G13A, which is known to facilitate a pronounced leakage of the target membrane prior to lipid mixing, reduces the membrane integrity by forming a ‘super’ bundle. Our simulations offer a new interpretation for a number of experimentally observed features of the fusion reaction mediated by the prototypical fusion protein, influenza hemagglutinin, and might bring new insights into mechanisms of other viral fusion reactions.

## Introduction

**Figure 1:**
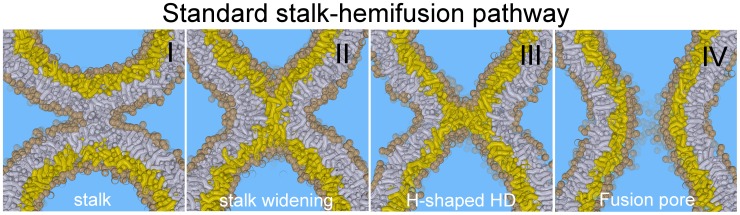
Standard stalk-hemifusion pathway (cross-section, side-view): The initial stalk (I) radially expands (II) forming an H-shaped hemifusion diaphragm (H-HD) after the trans-leaflets (colored yellow) meet (III). When the H-HD ruptures a fusion pore is formed (IV).

Membrane fusion is a fundamental process in biological cells, being involved in viral infection, endo- and exocytosis, and fertilization. The understanding of its molecular mechanism will open avenues for controlling a variety of collective biophysical processes that alter membrane topology. It is widely accepted that influenza hemagglutinin mediates a fusion mechanism that progresses through hemifusion [1], [2]. In the standard stalk-hemifusion pathway [3]–[5], illustrated in Fig. 1, the initial contact point between the apposing *cis*-leaflets, i.e. the stalk, progresses via an axially symmetric radial expansion (stalk widening), which thins the stalk such that the two distal *trans*-leaflets eventually meet and form a single-bilayer-thick H-shaped diaphragm (H-HD). After rupture of the H-HD, the fusion is completed.

Conductance measurements and fluorescence spectroscopy studies of fusion between the influenza envelope (host membrane) and lipid model membranes (target membrane) have reported that lipid mixing is preceded by the formation of an essential pre-fusion structure in the target membrane [6]–[11]. Formation of such a pre-fusion structure is believed to involve perforation of the target membrane [6], [7], because leakage through the target membrane is detected prior to lipid mixing [6]–[8], [11], [12].

**Figure 2:**
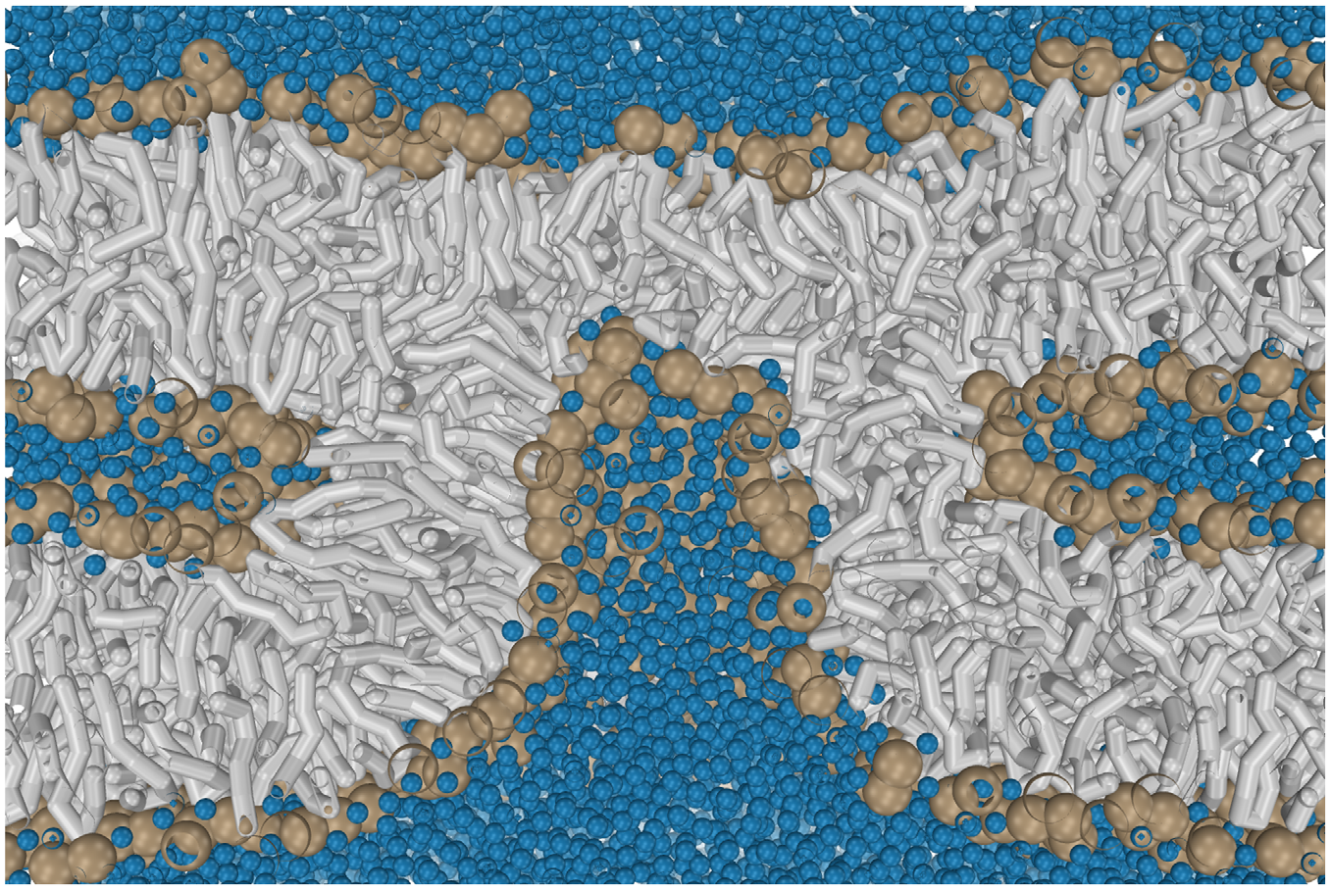
A 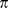-shaped hemifusion diaphragm (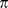-HD) which is generated by a stalk that has encircled a membrane pore.

Intriguingly, the formation of a small (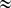
5 nm wide) stable 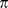-shaped HD (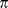-HD), illustrated in Fig. 2, which appears to be generated by a stalk that has partially encircled a formed membrane pore, has recently been observed by electron cryo-tomography of influenza fusion between a viral envelope and a pure DOPC vesicle [7]. It was reported that a leaky funnel-like structure was formed in the target membrane prior to lipid mixing. Moreover, in this example, the *trans*-leaflet of the viral membrane is completely covered by a rigid, shape-stabilizing protein matrix [7], that impedes bending of the *trans*-leaflets, which is required for the stalk to expand into an HD (Fig. 1 II) [4], [13]. Such limitation should have substantially increased the already large barrier to form an H-HD within the standard hemi-fusion mechanism, ranging from 15–63 k
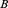
T in free membranes without protein matrix [4].

In model influenza fusion, single point mutations of the fusion peptide have been shown to either completely inhibit fusion, specifically arrest fusion at a hemifusion stage, or induce pronounced leakage of the target membrane *prior* to lipid mixing [11], [14], [15]. It is still puzzling why the fusion mechanism is so sensitive to minor changes in the fusion peptide.

The important philosophy behind point mutations is that they alter specific and essential peptide-membrane interactions and thereby modify the ability to overcome free-energy barriers in the underlying fusion mechanism of the wild-type peptide. Point mutation studies therefore play a key role in unraveling the mechanism of influenza fusion. It is reasonable to assume that, to stabilize the initial stalk, fusion peptides should stabilize the negatively curved stalk structure [3], [4], [13], [16]–[20]. Experiments, however, suggest that the wild-type peptide rather stabilizes neutral to weakly positively curved membrane structures, i.e. pores and dimples but not stalks [15], [21], [22]. The latter would suggest that the fusion peptides do not adhere to the negatively curved circumference of the stalk, which is supported by recent molecular simulations studies [23], [24]. If the peptides do not directly stabilize the stalk structure itself, it is plausible that they will promote the formation of alternate, highly stressed pre-fusion structures, such as a dimple and/or pore [6], [7], [16], that relax by both stabilizing and expanding the stalk in the course of fusion. Could the fusion inhibiting mutants G1V and W14A [14], [25] impair the formation of a required pre-fusion structure? Is the leakage of the target membrane observed prior to lipid mixing, as induced by point mutation G13A [11], possibly related to the corrupted formation of such pre-fusion structure?

Other point mutations facilitate hemifusion but selectively inhibit content mixing [11], [14], [15]. One of such intriguing point mutations is mutant G1S. In comparison with the wild-type, this mutant displays a similar secondary structure and structural dynamics and, in addition, also inserts similarly in the membrane [15]. It is difficult to conceive how the wild-type peptide, when adhering to the membrane outside of the virus or host cell [15], and when not being associated with the stalk structure [15], [21]–[24], would open the fusion pore. Is it possible that the terminal-hemifusion mutant G1S rather inhibits the fusion step prior to pore opening, i.e. the expansion of the stalk into a 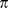-HD?

The aim of this work is to relate the three different experimentally observed phenomena in influenza fusion: the funnel-like pre-fusion structure, the 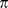-HD and the observed effect of the point mutations. We will demonstrate that these three phenomena can be understood from a fundamental and general concept in membrane physics: the line-tension, i.e. the free-energy per unit length [26], [27]. To this end, we use molecular simulation of a coarse-grained model [28], [29], where computational efficiency is enhanced by representing several atoms by a single interaction site. This description captures the underlying driving forces and evolution of the fusion process in near-atomic detail. Recently, this model has been successfully applied to study membrane fusion mediated by SNARE proteins [30] and long surfactant protein B [31]. To rationalize the above mentioned phenomena, we (i) study the physical properties of an isolated stalk formed between two apposing bilayers, (ii) investigate how these stalk properties are modified by the presence of a small hydrophilic pore in one of the bilayers, (iii) explore the relation between wild-type influenza peptides and stalk expansion, and (iv) study the effects of point mutations that are experimentally known to alter the peptide’s fusogenity.

## Results and Discussion

### Large Negative Spontaneous Curvature Results in Linear Stalk Elongation

**Figure 3:**
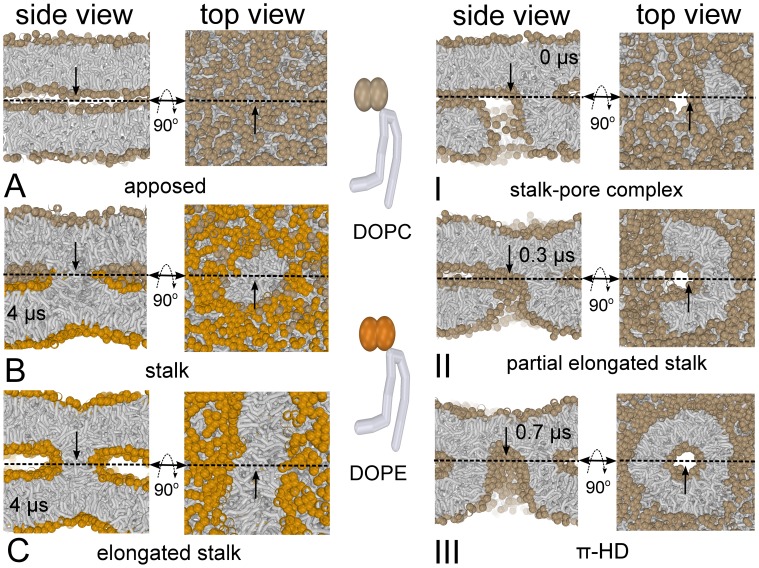
Evolution of a stalk in the absence and presence of a pore. For sake of clarity the size of the lipid headgroups is exaggerated (solvent is not shown). (A) Two apposed DOPC bilayers. A preformed stalk is not stable (see Fig. 4). (B) A stable ‘hour-glass shaped’ stalk structure formed between a DOPE and DOPC bilayer (4 µs). (C) Elongation of a stalk formed between two DOPE bilayers (4 µs). (I-III) Evolution of a stalk formed between two DOPC bilayers in the vicinity of a pore (stalk-pore complex). Elongation of the stalk, which circumvents the pore, results in the formation of a 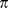-shaped hemifusion diaphragm (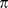-HD).

Fusion is believed to be initiated by the formation of an energetically costly negatively curved stalk structure [1]–[4], [13], [16]–[20]. To be able to rationalize the link between stalk and line-tension, we first study the expansion of an isolated stalk formed between two tension-less lipid membranes of area 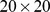 nm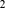 (1152 lipids each) that are separated by a 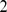 nm-thick water layer, corresponding to approximately 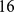 H
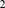
O molecules per lipid. After stalk formation, fusion has been proposed to progress by either a radial, axially symmetric expansion [3], [13], [16], [18] or, alternatively, by a linear elongation of the stalk [13], [19], [32], [33]. Lipids that are characterized by a large negative spontaneous curvature, e.g. DOPE lipids, form inverse lipid phases, e.g. the inverted hexagonal phase, at sufficiently low hydration and high temperature. The inverted hexagonal phase consists of an hexagonally ordered array of cylindrical bilayer structures. As observed in both, coarse-grained and atomistic simulations [34], [35], inverted hexagonal structures are formed by linear elongation of stalks between multiple stacked bilayers. This universal type of stalk instability characterizes the transition from the lamellar to the inverted hexagonal phase [36]. Such a linear elongation of a stalk is shown in Fig. 3 C for a stalk formed between *two* DOPE bilayers at 350 K. We estimated the line-tension 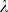, a measure for the free-energy per unit length, of such a linearly elongated DOPE stalk from the pressure tensor (see Fig. S1 for a detailed explanation) and obtained 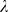 = −60
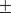
15 pN or about −14 k
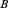
T per nm. A negative line-tension implies that it is thermodynamically favorable to increase the negatively curved perimeter of the stalk, i.e. stalk elongation. When we replace *one* of the DOPE bilayers by a pure DOPC bilayer, however, stalk elongation is inhibited and the initially formed ‘hour glass’-shaped (rhombohedral) stalk remains stable over the course of the 4 µs simulation (see Fig. 3 B). The latter effect can be explained by a positive value of 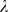 as a result of the increasing fraction of DOPC lipids, which have a more positive spontaneous curvature. Such asymmetric setup mimics the experimentally studied fusion between a pure DOPC membrane and the fusogenic viral envelope [7], where the occurrence of spontaneous stalk elongation presumably is not favorable [37], [38]. In such a case, a radial, axially symmetric stalk expansion, stipulated by the standard hemifusion mechanism, becomes favored over a linear expansion [13], [33]. This latter process, however, would face a substantial nucleation barrier that, depending on lipid polymorphism, has been estimated to be 15–63 k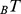 [4] and, indeed, such a radial stalk expansion is not observed in our simulation. Thus, in the absence of a sufficiently large negative spontaneous curvature, stalk expansion, either radially or by elongation, does not occur spontaneously [4], [13], [16], [18], [33], [39].

### The Presence of a Pore Results in the Formation of a 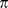-HD in Lamellar PC Bilayers

Alternative fusion mechanisms, which involve pores in one or both of the apposing membranes have been observed in computer simulation [5], [32], [40] and have been studied by self-consistent field (SCF) theory [33]. Next, we study whether the presence of an externally stabilized pore might overcome the free-energy cost of stalk elongation even for more symmetric, lamella-forming lipids. Since we focus on the stalk to HD transition, we do not investigate the formation of a stalk in the vicinity of a pore or *vice versa* but we start from the energetically favorable stalk-pore complex (Fig. 3 I) [32]. To this end, we consider that a stalk has been formed between *two* pure DOPC bilayers in the vicinity of a pore in the lower bilayer. Panels I-III of Fig. 3 show that the presence of a small 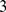 nm–wide pore, which is stabilized by an external field (cf. *methods*), is indeed able to facilitate stalk elongation even between lamella-forming DOPC bilayers at 310 & 350 K, and that such a process leads to the formation of a 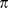-HD. Note that this process does not necessarily involve bending of the host membrane. Thus, the presence of a rigid, shape-stabilizing protein-matrix on the *trans*-leaflet of the viral envelope [7] would not impede such formation of a 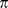-HD.

It is easy to rationalize that the observed effect has a simple essence. A pore generated in a lipid membrane, i.e. by membrane stretching or application of an electric field, has a rim covered by an extremely strongly curved lipid monolayer. The bending energy of this monolayer determines the rim’s line-tension. Sewing this rim to the host membrane replaces it with a circular three-bilayer junction, which is energetically favorable and leads to 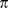-HD formation. We estimated the line-tension of the rim from the simulation of the corresponding bilayer edge (mimicking a pore with infinite radius) [41] and obtained 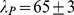 pN at 310 K. Indeed, the line-tension of a three-bilayer junction (the HD) was much lower, namely 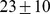 pN (see Fig. S1). This value is in qualitative agreement with values derived from continuum elastic models (15–20 pN) [13]. Elongation is favorable, and the metastable 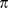-HD is formed, provided that the line-tension of the three-bilayer junction is lower than that of the replaced pore edge. Obviously, the topology of the fusion site resulting from such pore fusion (a 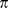-HD) is different from that generated by a dimple fusion (an H-HD) but the physical forces are the same.

**Figure 4:**
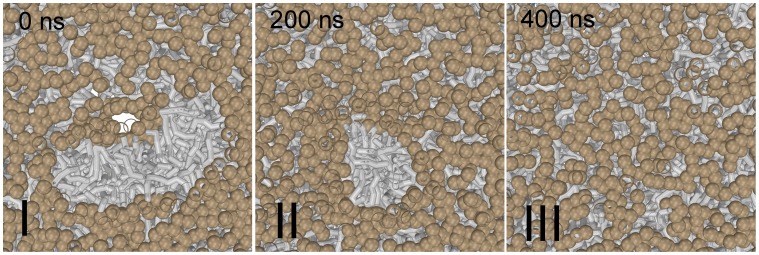
Stalk evolution in response of removing the pore (top view on porated bilayer). (I,II,III) Sudden removal of the pore *before* completion of the 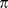-HD reverses the stalk elongation process, and the stalk completely disappears (DOPC at 310 K, with 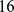 water molecules per lipid between the membranes). Hydrophobic lipid tails are colored grey, polar-headgroups (DOPC) tan.

Finally, to demonstrate that the observed linear elongation of the stalk crucially depends on the presence of a pore, we remove the pore when the stalk has slightly elongated. In such a case, the elongation reverses, and the stalk completely disappears (Fig. 4 I, II, III). Thus, in the absence of a pore, stalk structures are not stable in these lamella-forming bilayers (DOPC at 310 & 350 K, with 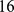 water molecules per lipid between the membranes), in qualitative agreement with X-ray studies [42]. The latter process might partly explain why lipid mixing in electrofusion is observed *after* formation of membrane pores rather than the opposite [43]. Hence, stabilization of the stalk under unfavorable conditions, e.g., a non-optimal temperature, membrane curvature [16], hydration level, or lipid composition, will necessarily require the presence of the pore. We additionally note that the disappearance of the stalk progressed through a similar intermediate as the one that has been observed in simulations of stalk formation [30], [44], [44], [45,45,46], i.e. a splayed lipid connecting the two adjacent leaflets (seeFig. S2).

In summary, these stalk-pore simulations suggest that stalk stabilization and expansion do not necessarily require the presence of lipids with a negative spontaneous curvature but can alternatively be facilitated by an external pore, whose curvature stress gives rise to a positive line-tension. There is, however, one conceptual difference: In our first example (Fig. 3 B & 3 C), the stalk or elongated stalk was stabilized by the presence of the intrinsic negatively curved DOPE lipids. In such a case, the DOPE lipids were the ‘lineactants’ [47], [48], i.e., molecules that favorably partition towards the stalk’s perimeter and thereby reduce its excess free-energy or line-tension. Likewise, also fusion peptides are often thought to be ‘lineactants’. In our second example, however, it was the stalk itself that was the lineactant, i.e. the stalk favorably partitioned toward the pore’s rim and reduced its line-tension. Could such inverted role, i.e. the stalk itself is the lineactant, explain influenza fusion?

### Influenza Fusion Peptides form a Trans-membrane Arranged Peptide Bundle that Drives Stalk Elongation

**Figure 5:**
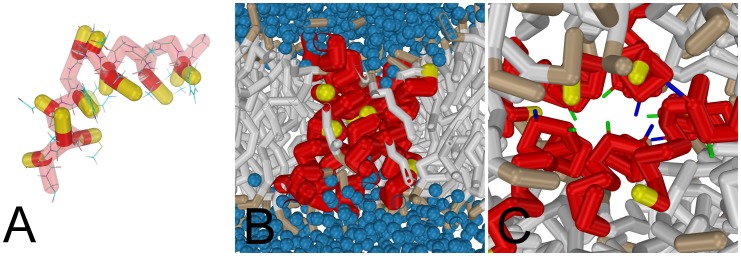
(A) Overlap between the coarse-grained model (backbone red and side-chains yellow) of the wild-type influenza fusion peptide and the NMR structure [**71**]. The two helices are joined by a linker region at a slightly bent angle (boomerang-shape). (B) The wild-type influenza fusion peptides (side-chains not shown) aggregate into a stable hexameric bundle. The bundle interior is depleted in solvent (colored blue) and lipid head groups. For sake of clarity, the first backbone residue (Gly1) is colored yellow. (C) Top view of the bundle. The bundle’s interior is mainly composed of the hydrophilic residues Glu11 (colored blue) and Asn12 (colored green) that are located in the kinked region of the peptide and which point toward the central axis of the bundle.

Motivated by the experimental observation of a 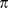-HD [7], and the hypothesis of the funnel structure [6], [7], [9], we next address the question of whether influenza peptides can, in principle, stabilize a ‘functional’ pre-fusion intermediate such as the pore shown in our previous example. To this aim, we included a near-atomistic coarse-grained model of the influenza hemagglutinin fusion peptide [23], [24]. This model accurately reproduces the general structure of two helices joined by a linker region at a slightly bent angle (Fig. 5 A) [15], [25]. In addition, it successfully mimics the amphiphilic nature of the influenza hemagglutinin fusion peptide. Intriguingly, we observed that these fusion peptides possess a strong propensity to self-associate into 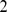 nm-wide trans-membrane arranged bundles consisting of 4–6 peptides (Fig. 5 B & *methods*). These bundles were found to be stable in the course of a 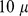 s-lasting simulation. Such a peptide bundle conceptually differs from the externally stabilized hydrophilic pore that we studied in our previous example. The internal rim of the peptide ‘pore’ is lined with the amphiphilic peptides that replace the lipid head groups and solvent. The bundle’s interior is mainly composed of the hydrophilic residues Glu11 and Asn12, which point toward the central axis of the bundle (Fig. 5 C). This particular region in the peptide, i.e. the kink region, has been shown to play a major role in the peptide’s fusogenicity [11], [15], [25]. Such bundle structure is, in fact, similar to the hexameric bundle formed by the related parainfluenza virus 5 (PIV5) fusion peptide as observed in recent atomistic simulations [9]. Such pore structure seems essentially non-leaky and therefore rather resembles the closed structure of the mechanosensitive channel protein MscL [9] than a hydrophilic toroidal pore formed by antimicrobial peptides [49].

Although the peptide bundle is a self-stabilized structure, this does *not* imply that the lipid rim that surrounds the bundle is characterized by a vanishing excess of free-energy [50], [51]. Assigning this quantity to the bundle circumference, we define its line-tension. Whereas the line-tension of the hydrophilic pore in our previous example was counter-balanced by an externally applied potential, the line-tension of the rim of the peptide bundle stems from a balance between the remaining hydrophobic mismatch between peptides and lipids, and the short-range repulsions (excluded volume) between the densely packed peptides in the bundle. A non-vanishing positive line-tension is important: (i) It can both stabilize and promote the expansion of the stalk, and (ii) it favors minimization of the bundle’s perimeter and thereby exerts a constricting force on the peptide bundle that ensures formation of a closed, essentially non-leaky structure. If the peptide bundle were an in-vivo relevant fusion intermediate the latter would be important. Hence, like synaptic fusion, viral fusion is generally believed to proceed via a non-leaky process, because excessive leakage might harm the host cell or virus. The stability condition of the peptide bundle/pore further implies that the effective, attractive medium-ranged peptide-peptide interactions, which are gained when the bundle is assembled, *exceed* the induced line-tension.

**Figure 6:**
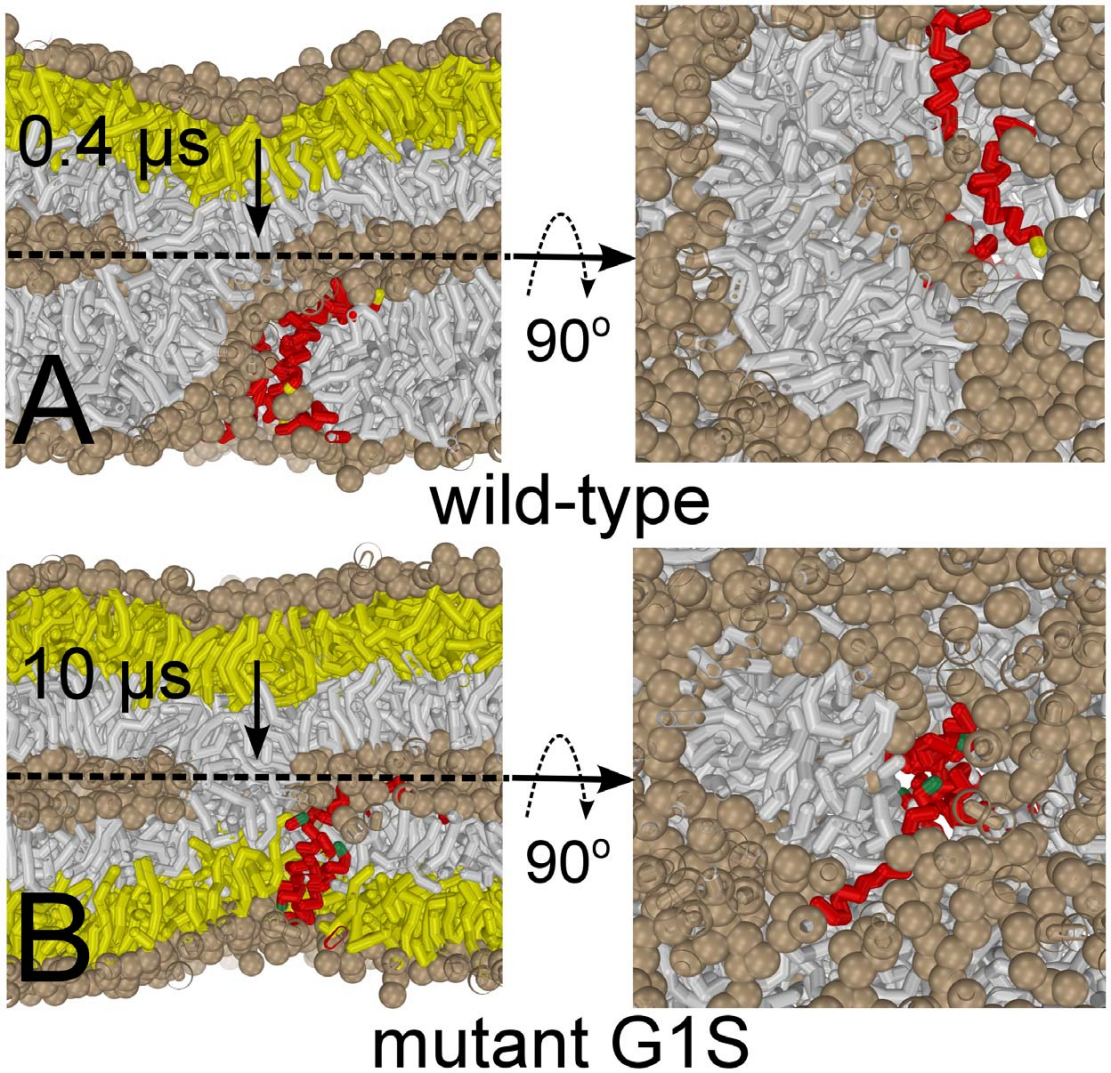
Evolution of the stalk in the presence of the peptide bundle. For sake of clarity the size of the lipid headgroups is exaggerated (solvent is not shown). (A) The elongated stalk (wild-type peptides) after 0.4 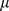
s. The bundle has opened up and the stalk and has partly surrounded the formed hole. Notice the readily adopted banana-shape. The stalk forces the peptides to the remaining rim portion. At this stage mixing occurs between both the *cis*-leaflets and the *trans*-leaflet of the target membrane (colored gray), while the *cis*-leaflet of the host cell (colored yellow) does not contribute to lipid mixing. (B) Mutating a single residue in the peptides, Gly1 to Ser1 (colored green), known as the terminal hemifusion mutant G1S [14], [25], stabilizes both bundle and stalk but inhibits elongation of the stalk (10 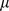
s). Consequentially, the fusion reaction becomes trapped. Note that lipid head-groups are excluded from the pore interior and the *trans*-leaflets (colored yellow) are hindered from participating in the lipid mixing.

We simulated the fusion reaction between the two DOPC membranes in the presence of the peptide bundle and explored if such bundle can, in principle, stabilize the stalk and drive its subsequent expansion. In 5 out of 5 simulations, stalk elongation occurred as a nucleated event [32], [33] within 4 µs. Fig. 6 A depicts such elongation of the stalk in the presence of the peptide bundle (see Fig. S4, for additional simulations). It appears that the elongating stalk does not surround the bundle but rather ‘opens’ the closed bundle structure and pushes the peptides away. Such nucleated opening of the bundle indicates that a stalk formed near the intact bundle (Fig. 6 B) is under high stress, and vice versa. As the stalk elongates, the free rim portion of the resulting stalk-pore complex [33], which is not surrounded by the stalk, shrinks and the peptides crowd in this location (Fig. 6 A). The elongated stalk stabilizes a hydrophilic rim which is composed of lipid head groups (i.e, the 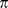-HD). In contrast, the free portion of the rim which is stabilized by the peptides remains hydrophobic. Because the peptides are forced in close contact with the ends of the stalk, they might partly stabilize these ends by lowering their Gaussian curvature energy [23], [24], [33], [38]. The replacement of the peptides by the stalk at the rim of such a pore further indicates (i) a non-vanishing positive line-tension, (ii) a pronounced difference of line-tension between its rim and the 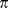-HD, and (iii) that the stalk is the better lineactant, i.e. it has a higher affinity for its rim than the peptides.

To conclude, our simulations suggest that the wild-type influenza fusion peptide can in principle form a functional pre-fusion intermediate [6], [7], [9], i.e. a bundle consisting of multiple trans-membrane arranged fusion peptides, and that such an intermediate can facilitate the subsequent formation of the experimentally observed 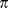-HD [7].

### Point Mutations Affect Stability and Fusogenicity of the Peptide Bundle

The relation between the peptide bundle and fusion is further corroborated by studies of the influence of peptide mutations. We discuss four specific examples:

(I) A point mutation (G1V) where the first residue in the peptide, glycine, is replaced by valine, completely inhibits membrane fusion (lipid mixing) [14]. When adhered to the membrane surface, the fusogenic wild-type peptide adopts a boomerang shape (cf. Fig. 5 A), while the non-fusogenic G1V mutant remains linear, 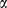-helical [15]. It has been demonstrated by atomistic simulations that such linear 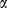-helical structure strongly reduces the peptides ability to penetrate the hydrophobic membrane core [15], i.e., the peptides remain parallel to the membrane surface. It is intuitive that the inability of the G1V mutant to adopt a trans-membrane orientation will also affect the formation/stabilization of the observed peptide bundle.

**Figure 7:**
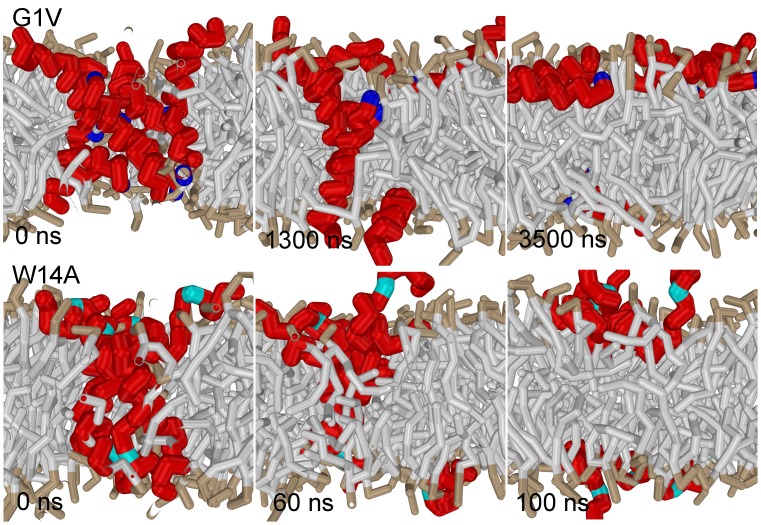
Point mutations that are known to inhibit fusion destabilize the bundle. (top) Mutating a single residue, Gly1 to Val1 (colored blue), destabilizes the peptide bundle (mutant G1V). (bottom) Mutating a single residue, Trp14 to Ala14 (colored cyan), rapidly destabilizes the peptide bundle (mutant W14A). Notice the flexible kink that points out of the membrane [25].

We have performed simulations of *wild-type* peptides forming a stable bundle (see *methods*). Then, the first residue, glycine, was replaced with valine together with adapting the secondary structure to the linear 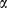-helical structure [15]. Such point mutation rapidly destabilized the bundle in 5 out of 5 simulations (Fig. 7). Thus, our simulations indicate that the G1V mutation indeed impairs the ability of the peptides to form stable bundles. We found this effect, however, to be independent of secondary structure. Bundle destabilization also occurred when we conserved the secondary structure of the wild-type. In contrast, the bundle remained stable in the presence of fully linear, 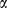-helical wild-type peptides. We therefore relate the destabilization of the bundle to the increased hydrophobicity of the first residue rather than the concomitant change in secondary structure. This effect can be rationalized by the reduced ability of the hydrophobic Val1 residue to line-up with both the lipid/solvent interface and the hydrophilic center of the bundle (Fig. 5 B & C). However, the boomerang shape of the wild-type [15], [25] might help the peptides to more easily adapt the trans-membrane orientation that is required to form such bundle. Although the G1V mutation will also affect other functions of these peptides related to fusion, e.g. the formation of the dimple [7], [52], the lipid protrusion frequency [46], solvent dynamics [53], or membrane curvature/elasticity [38], the important role of the bundle in the viral fusion process indicated by our simulations suggests that the absence of a bundle substantially affects both the stability of the initial stalk and the pathway of the following fusion reaction. Aside from stabilizing the stalk structure, a bundle also enhances formation of the stalk by allowing a closer proximity between the adjacent fusion sites [39], e.g., by locally perturbing the membrane structure and reducing the inter-membrane repulsion (see Fig. S3). If viral fusion in model systems proceeded only after the formation of an initial ‘funnel’ structure in the target membrane [6], [7], [9], the non-fusogenicity of the point mutation could be rationalized by its inability to induce this early step of the fusion process.

(II) Similar to mutant G1V, also mutant W14A has been shown to inhibit lipid mixing [25]. The structure of W14A determined by NMR and site-directed spin labeling features a flexible kink that points out of the membrane, in sharp contrast to the more ordered boomerang shape of the wild-type, which points into the membrane [25]. Although the flexible structure of mutant W14A rather differs from the conserved linear 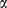-helical structure of mutant G1V, we expect that also mutant W14A destabilizes the bundle because it shares the inability of mutant G1V to induce fusion. To this end, we modeled mutant W14A based on its NMR derived structure (cf. *methods*) and studied its ability to stabilize the bundle. Figure 7 shows that mutant W14A destabilizes the bundle within 100 ns. Thus, the inability to stabilize the bundle seems common to both of these structurally different point mutations.

(III) Another well known and intriguing point mutation of the peptide, Gly1 to Ser1 (mutant G1S), facilitates lipid mixing but selectively impairs its ability to complete fusion, i.e. content mixing [14]. Combined NMR and atomistic simulation studies have shown that this G1S mutant adopts a similar boomerang shape, displays similar structural dynamics, and inserts similarly in the hydrophobic bilayer core as the bundle-forming wild-peptide [15].

In contrast to mutant G1V, mutant G1S does not destabilize the bundle over the course of all 5 simulations lasting 10 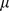
s. Thus, our simulations predict that also the G1S mutant is able to stabilize a peptide bundle. The latter might be explained by the fact that, unlike valine (mutant G1V), serine is even slightly more hydrophilic than glycine (wild-type), which makes lining up with both the hydrophilic center of the bundle and the lipid/solvent interface energetically favorable. Thus, we assume that the G1S mutant forms a stable bundle and, according to the here-proposed stalk-bundle mechanism, such a bundle will, in turn, facilitate the formation/stabilization of a stalk. This stalk-bundle formation is corroborated by the experimentally observed lipid mixing [14]. How can this situation be reconciled with the peptide’s inability to complete fusion? To this end, we investigated the behavior of a stalk-bundle complex formed between two DOPC bilayers. We observed that the presence of the G1S mutant bundle stabilizes the stalk in the course of the simulation of 10 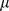
s but, in contrast to the bundle of wild-type peptides, the stalk does not ‘open’ the bundle and lines the interior pore (5 out of 5 simulations). This first observation suggests that the stresses imposed on the lipid rim of the preserved bundle are either too small and/or the affinity between the peptides in the bundle is too large. In other words such closed bundle structure seems too favorable/stable with respect to the elongated stalk. Since stalk elongation seems to be a nucleated event that requires an initial ‘opening’ of the bundle (Fig. 6A), the presence of a persistent bundle opposes stalk elongation.

**Figure 8:**
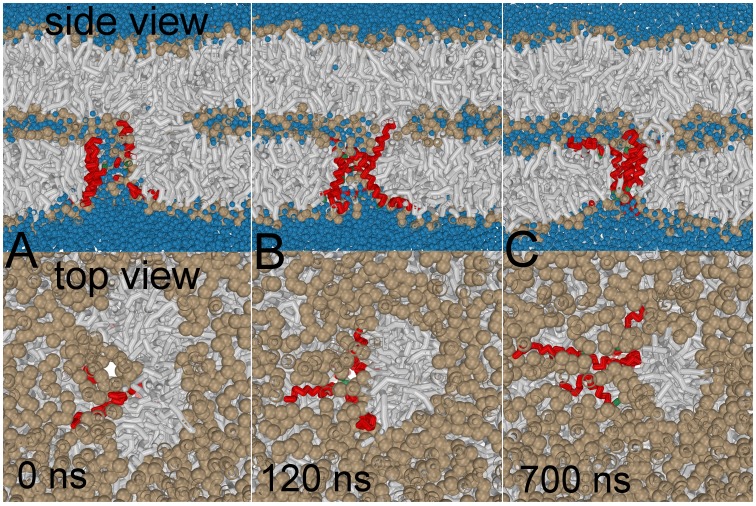
The G1S mutation reverses the stalk elongation process facilitated by the wild-type. Notice the removal of solvent (colored blue) and lipid head-groups (colored tan) from the membrane interior when the peptide bundle ‘reseals’ itself – the stalk and peptide are competitive lineactants.

To further explore the stability of such bundle against stalk elongation, we took a corresponding wild-type simulation where nucleation of the elongation process had readily occurred, and ‘on the fly’ mutated the wild-type into the G1S mutant. Intriguingly, such mutation actually reversed the elongation process and recovered the bundle (Fig. 8). This reversibility indicates that the G1S mutation in fact makes stalk elongation a continuous energetically ‘uphill’ process rather than a nucleated event, and illustrates that the peptide and the stalk are competitive lineactants – a hydrophobic lipid rim is a prerequisite for the peptide/bundle, whereas a hydrophilic rim is a prerequisite for the elongated stalk (Fig. 8). Apparently, the G1S mutant is the better lineactant, i.e. it has the higher affinity for the lipid rim. The inability of the stalk to elongate and form a 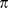-HD imparts a higher barrier onto the fusion process, which traps the membranes in an incomplete fusion state in our simulation as well as in experiments [14]. We note that such a trapped incomplete fusion state facilitates mixing predominantly between the *cis*-leaflets, similar to the standard stalk-hemifusion pathway (cf. Fig. 1 A) because lipid head-groups are excluded from the interior of the preserved bundle structure and thereby hinder the occurrence of flip-flops from the *trans*- to *cis*-leaflets (Fig. 6B).

(IV) The last point mutation of interest is the recently discovered mutant G13A [11]. Whereas the wild-type peptide has a steep kink-angle of 105 degrees, mutant G13A has a shallower kink-angle of about 150 degrees [11]. Fluorescence microscopy studies have observed that when red blood cells expressing such mutant merge, the content of the cells is released into the inter-cellular space rather than being transferred between the fusing cells [11]. Such massive leakage is readily observed more than 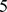 minutes *before* the occurrence of lipid mixing [11]. Leakage of the target membrane prior to lipid mixing, albeit less pronounced, has also been observed in model influenza fusion with wild-type peptides [6], [7], [12].

Notably, there seems to be a close relationship between a peptide’s ability to induce membrane lysis (leakage) and its ability to induce fusion. Antimicrobial peptides such as melittin, which are primarily pore-forming, have been shown capable to induce fusion [54], [55], whereas fusion peptides, such as influenza hemagglutinin, have been shown capable to induce membrane lysis [56]–[59]. Furthermore, it has been observed that deletion of the N- or C-terminus of the influenza fusion peptide or its mutants, decrease its ability to induce both lysis and fusion [56], [57]. With respect to the here-proposed stalk-bundle mechanism, such a relationship is intuitive because both fusion and lysis relate to the line-tension and the peptides are lineactants in both cases.

**Figure 9:**
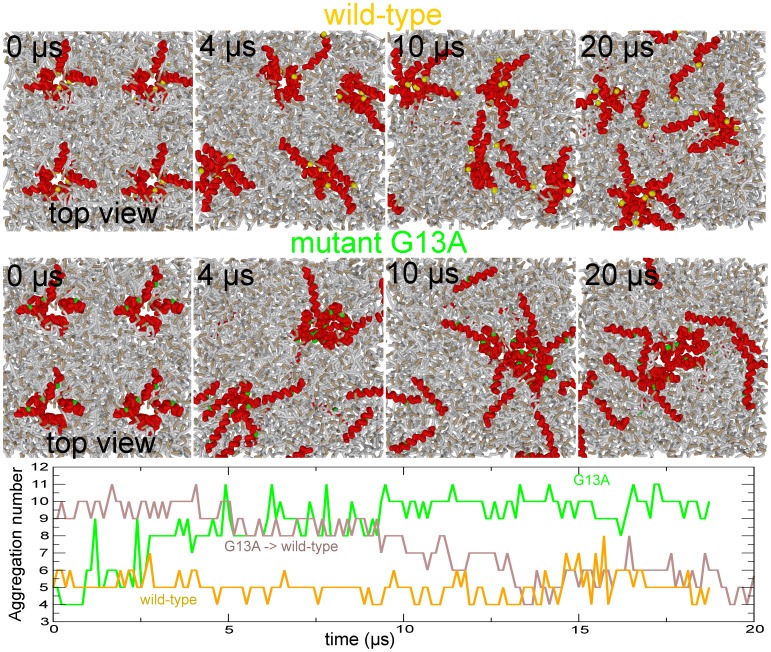
Interaction between multiple peptide bundles. (upper panel) Four wild-type bundles (top-view). The bundles strongly repel each other and maximize their separation distance in the course of the simulation. Eventually one of the bundles vanishes. (middle panel) Four G13A mutant bundles. The bundles are attractive and their coalescence results in a ‘super’ bundle consisting of 10 trans-membrane arranged peptides. (lower panel) Aggregation number of the largest bundle in the course of the simulation (Only the trans-membrane arranged peptide are counted). The brown line shows a separate simulation where the G13A mutation is reversed after 20 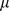
s (G13A -> wild-type). The wild-type ‘super’ bundle readopts its usual size in the course of the simulation.

**Figure 10:**
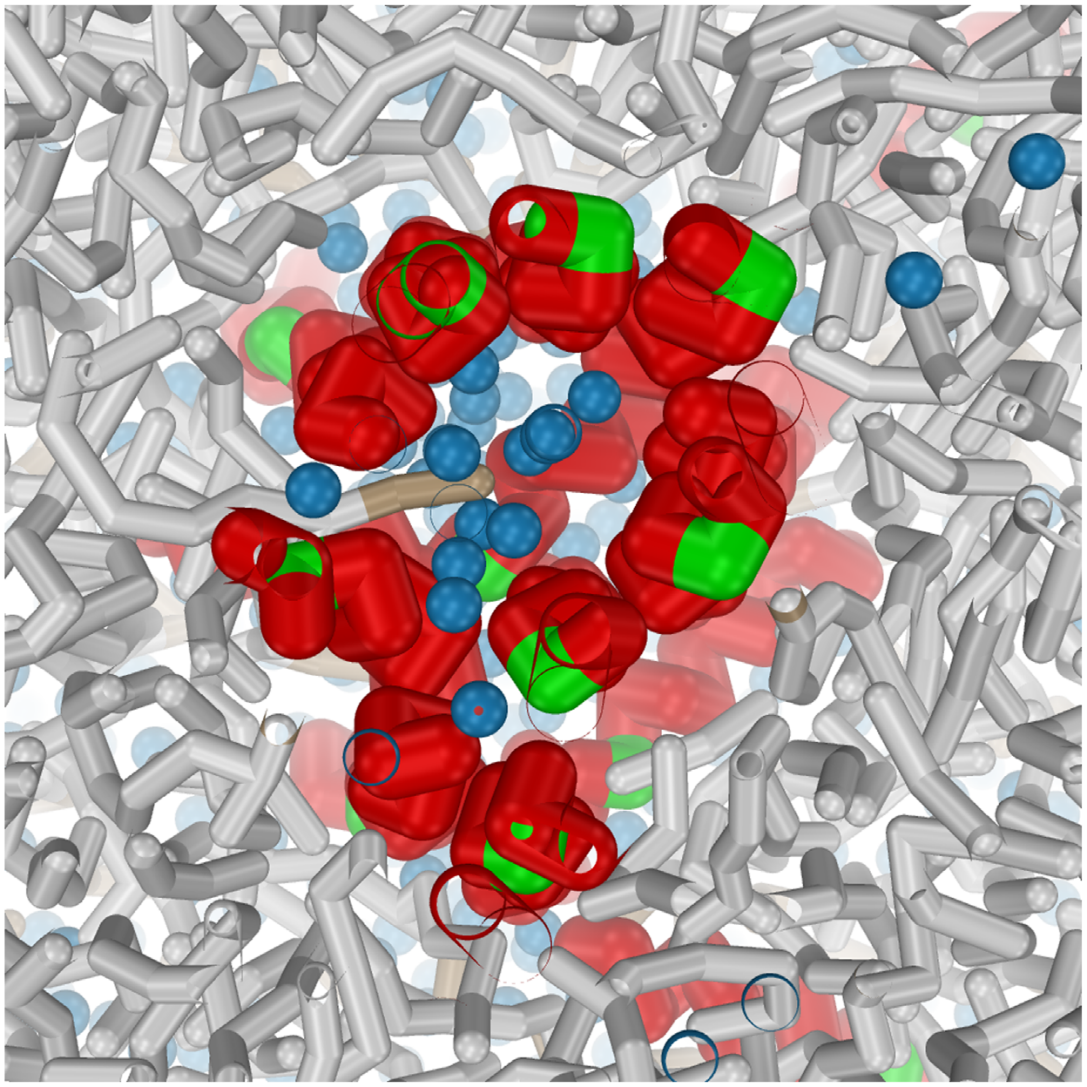
Detailed view of the ‘super’ bundle (10 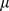
s) formed by the leaky fusion mutant G13A (Top view, cross-section through the bilayer center). Notice that residue 13 (colored green) directly faces the hydrophobic lipid rim around the bundle. The solvent (colored blue) in the center of the bundle suggests the occurrence of leakage prior to lipid mixing [11].

We explored the behavior of the bundles formed by mutant G13A. To do so, we positioned 4 bundles, each consisting of six trans-membrane arranged peptides plus two ‘bystander’ peptides, in a 15×15 nm POPC bilayer. We also duplicated this setup for the wild-type bundles. Fig. 9 shows both setups. In the course of the 20 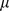
s simulation we observed a strong repulsive behavior between the separate wild-type bundles, i.e. the bundles tend to maximize their separation distance (Fig. 9, upper panel). Eventually, one of the bundles breaks up and the peptides redistribute among the remaining bundles. The size of these bundles ranges from tetrameters to hexamers, with pentamers being the most abundant (Fig. 9, lower panel). Notably, also the surface adhered peptides display an interaction with the bundle and likely play a role in its stability. In contrast to the wild-type bundles, the bundles formed by mutant G13A are ‘attractive’ and coalescence between the different bundles is observed (Fig. 9, central panel). Eventually, the latter results in *one* large bundle consisting of 10 trans-membrane arranged peptides and several associated bystander peptides. Obviously, the formation of such a ‘super’ bundle would be ominous for the integrity of the membrane (Fig. 10), especially in the presence of additional stress. Thus, the action of mutant G13A, i.e. massive leakage prior to lipid mixing, is very well explained by the formation of a corrupted peptide bundle. How can we rationalize this effect? We hypothesize that such effect stems form altering the Janus-like structure of the peptide, i.e. their mutual interactions and their hydrophobic mismatch with the lipid membrane. The Janus-like structure of the peptides and the concomitant directionality of their mutual interaction is very important. If the peptides were merely axially symmetric cylinders, the balance between their mutual interaction and the hydrophobic mismatch (i.e. the interaction with the lipids) would likely dictate clustering or absence thereof [51]. In order to obtain a well-defined finite aggregation number one needs either a special geometry of packing or a special geometry of the interactions. The increased hydrophobicity of residue 13 (Gly13 to Ala13), which directly faces the lipid rim (Fig. 10), and the concomitant shallower kink-angle of the peptide most likely changes the optimal packing within the bundle and thereby drastically increases the aggregation number. In support of these arguments, we note that the ‘super’ bundle spontaneously reduces to its normal size *after* reversing the G13A mutation (Fig. 9, lower panel). For a lineactant, there appears to be a thin line between acting like a functional fusion peptide or antimicrobial peptide [60]. The G13A mutant, however, seems to behave more like an antimicrobial peptide.

In summary, our molecular simulations and free-energy arguments in conjunction with experimental observations suggest that the influenza fusion mechanism might proceed through the formation of a trans-membrane arranged peptide bundle in the target membrane [6], [7], [9]. We have shown that the lipidic rim around a pore or peptide bundle is a critical factor in membrane fusion, equivalent to membrane curvature, the commonly cited determinant. In fact, curvature stress and line-tension are closely related quantities in membrane physics. For example, in the process of domain budding, minimization of the unfavorable perimeter of raft domains drives membrane bending (budding) and formation of vesicles (fission) [61]. Our simulations further suggest that ‘fusogenic’ peptides strike a balance: On one hand, they induce or stabilize a bundle in one of the membranes by gaining relatively favorable peptide-peptide and peptide-lipid interactions. On the other hand, the affinity of the peptides to pack into a bundle should be smaller than the stalk’s affinity for the lipid rim and the concomitant line-tension of the lipid rim has to be large enough in order to allow the stalk to surround it and form a 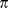-HD. In addition, the peptide bundle should keep a low aggregation number. Such a complex and subtle balance might very well explain the experimentally observed sensitivity of the fusion process towards point mutations in the influenza fusion peptide.

Based on our simulations, we predict that the puzzling trapped hemifusion state formed by point mutants G1S and E11A [14], [25] is a kinetically trapped stalk that forms a stable complex with the peptide bundle. Notably, the transmembrane domain of influenza hemagglutinin (TMD) is also known to be involved in the formation of the fusion pore [62]–[65]. In SNARE-mediated membrane fusion such pore formation seems facilitated by the end residues (C-termini) of the TMD, which are being pulled through the HD [30], and thereby actively open the fusion pore (see [39] for a recent review). A similar scenario may apply to influenza fusion. If the fusion peptide indeed facilitate the fusion process up to the formation of the HD, with the C-terminus of the TMD being chiefly involved in the subsequent pore opening, this would explain why the influenza fusion mechanism is relatively insensitive to overall changes in TMD sequence or length [62]–[65]. In such a case, the ability of hemagglutinin to complete fusion mainly relates to the ability of the C-terminus to perforate the membrane (HD). The latter would, e.g., be affected by altering the hydrophobicity, electrostatics or size of the TMD C-terminus and/or cytoplasmic domain [66]. We predict that the trapped hemifusion state that is due to substantial shortening of the hemagglutinin TMD [62], [63] or mutations herein [64]–[66], is a kinetically trapped 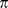-shaped hemifusion diaphragm. Future cryo-tomography studies should be able to resolve these different hemifused structures.

In this work we have mainly focused on how the peptides stabilize the stalk and drive its expansion into the hemifusion diaphragm. We have thus far not discussed the process of stalk formation itself. Molecular simulations suggest that stalk formation is facilitated by the formation of splayed lipid intermediates (i.e., the stalk barrier) which form a bridge between the apposed leaflets [30], [44]–[46]. Recent coarse grained and atomistic simulations have revealed that the wild-type peptide induces a substantial disorder in the packing of the lipid tails (tail protrusions) and thereby substantially reduces the membrane thickness [22], [46], [67]. The non-fusogenic mutants, however, show a less pronounced distortion of the membrane integrity [22]. The absorbed wild-type peptide induces an approximately 5-fold increase in the normal tail protrusion frequency [46], i.e. a tail-end reaches the lipid head-group plane five times more often per time interval. If these protrusions would enhance stalk formation *directly* via the formation of splayed lipid intermediates, they are expected to lower the apparent stalk barrier by, ln(5)k
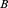
T = 1.6 k
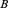
T [46]. Notably, a larger peptide:lipid ratio likely enhances such effect [67]. However, because of the logarithmic dependence, in order to lower the stalk barrier, by e.g. 15–40 k
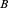
T, one would require an 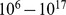 fold increase in the total protrusion frequency. Thus, it seems rather unlikely that the wild-type peptide facilitates stalk formation directly via tail protrusions. In addition, it is hard to reconcile how protrusions, when only being involved in stalk formation, would explain the action of the terminal hemifusion mutants G1S/E11A and the leaky fusion mutant G13A. Tail protrusions, or equivalently membrane thinning, reflect the presence of stress. Apparently, the adsorption of the wild-type peptide induces a substantial stress in the membrane. For a surface adhered amphiphilic peptide, however, the ability to induce stress is limited because the membrane will alternatively respond by forming pores or trans-membrane arranged bundles (i.e., when the peptides are lineactants) [50], [51]. In addition, such building up of surface stress causes competitive bending of the membrane [68]. Thus, the peptide-induced stress, i.e. the observed protrusions/thinning, might also facilitate other processes such as bundle, pore and dimple formation [7], [52].

With the dimple being hypothesized as a highly fusogenic and thus very transient intermediate in membrane fusion, one would not expect its direct observation. However, the observation of (intact) dimples by cryo-electron microscopy in wild-type influenza fusion [7] suggests that these dimples are in fact long-lived states with a slow escape rate – they are quite resistant to fusion. We emphasize that the free-energy of the stalk intermediate, i.e. the stalk barrier, mainly depends on the distance between the opposing leaflets and is rather independent of the leaflet’s curvature [20], [39], [69]. Thus, the important role of the dimple is to bring the leaflets into close proximity. To this aim, a dimple lowers the energetic cost of leaflet approach, i.e. the inter-membrane repulsion, both by reducing the effective contact area of the fusion site and by increasing its surface hydrophobicity (i.e., curvature stress) [39]. Its direct observation, however, suggests that the peptide-coated dimple might not suffice in bringing the membranes sufficiently close. In these experiments [6], [7], fusion was only observed *after* formation of a funnel-like intermediate in the target membrane. Notably, a bundle allows an additional proximity by tilting the membrane and such a ‘volcano’ structure substantially eases stalk formation (see Fig. S3).

To this end, our simulations offer a new interpretation for a number of known features of the fusion reaction mediated by the prototype fusion protein, influenza hemagglutinin, and might bring new insights into mechanisms of other viral fusion reactions. We hypothesize that such a line-tension controlled fusion mechanism, which closely resembles the mechanism of electrofusion [43], might be the direct evolutionary consequence of the substantial free-energy barriers [4], [5], [27] that the viral fusion machinery faces in its attempt to fuse with membranes that are, in turn, evolutionary designed to be largely resistant to (viral) fusion.

It is a great pleasure to thank Michael Schick, Reinhard Jahn, Leonid Chernomordik and Volker Knecht for stimulating discussions and constructive comments. Financial support has been provided by the DFG under grant SFB 803/B2-B3.

## Methods

### Pore- and Stalk Formation

The hydrophilic pore of radius 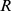 = 2.0 nm, located in one of the bilayers, was stabilized using an repulsive potential 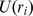 with 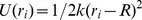, if 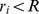 and 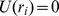 if 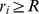, where r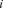 denotes the distance of the center of mass of the lipid from the pore center and 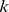 a force constant (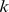 = 50 kJ nm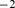 mol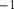) [41], [70]. Likewise, we induced the initial stalk in the bilayer fusion setup by applying an external field. Here, we applied the same harmonic potential to induce a 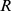 = 1.0 nm ‘void’ in the solvent layer between the bilayers. The hydrophobic nature of the void attracts the lipid tails in the adjacent leaflets and results in the formation of a stalk. After the stalk formation, the external field has been removed to allow for an additional 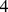 ns equilibration of the stalk structure.

### Peptide Model

All peptides used consisted of 20 aminoacids: GLFGAIAGFIENGWEGMIDG (wild-type). The Martini coarse-grained model [28], [29] captures the specific nature of each individual amino acid but does not predict secondary structure. The secondary structure and protonation state of the wild-type peptide (pdb:1IBN) and mutants (pdb:1×OP, pdb:2DCI, pdb:1×OO, pdb:2L4G) were derived from the NMR-resolved structures [15], [71]. The secondary structure was modeled by both restraining proper dihedrals between four neighboring backbone beads with an harmonic potential and by altering the non-bonded interactions according to the imposed secondary structure (free in solution, or in a coil or bend the backbone has a more polar character than in a helix or 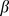-strand). Further details concerning this methodology can be found in the original publication [29]. To reproduce the experimentally observed ‘fixed’ angle between the two helices (the boomerang shape) [15], [22], [71] additional dihedral angle potentials were explicitly introduced for the backbone beads of residue 8 to 16. Further details are given in Ref. [23]. Recent atomistic simulations suggested that the ‘kink’ is in fact less preserved in the G1S mutant than in the wild-type [22]. We corrected for the latter effect by allowing a slightly larger flexibility between the two helices (i.e., without the additional dihedral restraints). For mutant G13A, an additional elastic network (Force constant of 500 kJ nm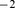 Mol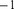) between the nearest backbone beads (cutoff between 0.5–0.9 nm) was applied to conserve its shallow 150 degrees tilt angle. Atomistic simulations of the wild-type in an implicit membrane environment suggested that the NMR-derived kink (secondary structure) is altered when the peptides closely pack into an trans-membrane arranged bundle/oligomer [72]. Although such prediction is beyond the capability of our model, we emphasize that both a linear and kinked wild-type peptide stabilizes the bundle in our simulations (see the section about mutant G1V), whereas a linear or kinked G1V mutant does not.

### Peptide Bundle

Stable peptides pores were formed by simulating 8 peptides, adhered to the membrane surface of a 10
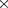
10 nm POPC bilayer (128 lipids), in the presence of externally stabilized hydrophilic pores with a diameter of 2–3 nm (see above). The peptides (both wild types and mutants) showed a remarkable attraction towards the pore’s rim. The external pore-stabilizing potential was removed after all peptides were assembled into the pore. We found that at least 4 peptides were required to form self-stabilized bundles. We additionally tested such scenario in pure DOPC, pure DOPE and pure POPE bilayers and found that the formed bundles were stable in all cases. Furthermore, these wild-type bundles were found to be stable at both 310 and 350 K in the course of the 10 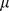
s simulation (in total about 20 simulations were preformed). In addition, we also tested the stability of the wild-type bundle with all the peptides oriented in the same direction, and the stability of the bundle in the polar Martini forcefield [73] in the presence of long-range electrostatic effects (PME). In all cases the bundle was found to be stable.

To investigate the stability of the mutant bundles, the G1S, G1V, and W14A mutations were performed on the stable and equilibrated wild-type bundle (at 350 K). Statistics was collected by performing multiple simulations (5 for each mutant) with different starting velocities.

### Bundle-facilitated Stalk Elongation

The bundle-facilitated stalk elongation was performed within the same system as the hydrophilic pore facilitated stalk elongation (DOPC, 1152 lipids per membrane, 16 water molecules per lipid within the inter-membrane space). Here, a pre-formed and equilibrated bundle was carefully embedded in a constructed pore and was additionally equilibrated for 40 ns. Then the stalk was induced according to the procedure described above. To be able to capture the bundle-facilitated stalk elongation (which is a nucleated event) within the limited time scales of the simulation, the simulations were performed at a slightly elevated temperature of 350 K (instead of 310 K). We emphasize that the absence of the bundle or pore also destabilizes the stalk at 350 K.

The G1S mutation was performed on the readily equilibrated ‘stalk-bundle’ setup of the wild-type. Statistics was collected by performing multiple simulations (5 with for each) with different starting velocities.

### Coalescence of the G13A Bundles

Here, a POPC membrane (128 lipids) with an equilibrated wild-type bundle (consisting of 6 trans-membrane arranged peptides and 2 ‘bystander’ peptides) was copied in both the X and Y dimension. The G13A mutation was performed on this system. Both wild-type and G13A mutant simulations were run at 310 K. For the reverse simulation (G13A to wild-type) the final snapshot of the G13A simulation was taken, and the G13A mutation was restored to the wild-type.

The peptide aggregation number of the largest bundle was calculated using a cluster algorithm. This algorithm clustered all back-bone atoms, which where located within +1.0 and −1.0 nm from the membrane center, based on a distance cutoff of 2.0 nm.

### Simulation Details

The simulations described in this paper were performed with the GROMACS simulation package [74], version 4.0.5. We used the Martini model version 2.1 [28], [29] to simulate the lipids and amino acids. In all simulations the system was coupled to a constant temperature bath [75] at 310 or 350 K with a relaxation time 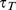 of 1.0 ps. The time step used in the simulation was 20 fs [76]. Shifted potentials were used to describe van der Waals and electrostatic pair-wise interactions. In both cases, the neighbor list cutoff was 1.2 nm and these potentials were gradually shifted to zero when the pair-wise distance exceeded 0.9 nm (van der Waals) or 0 nm (Coulomb). The neighbor list was updated every 10 simulation steps. The pressure was weakly coupled [75] to 1 bar with a relaxation time 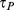 of 0.5 ps. In analogy to the other studies employing the Martini model, time scales quoted in this work were scaled by a factor of 4 to correct for the 4-times faster diffusion rates of water and lipids in the coarse-grained model [28] with respect to reality.

## Supporting Information

Figure S1 **Calculation of the line-tension of the elongated stalk and HD.**
(TIF)

Figure S2 **Splayed lipid intermediate observed before the stalk disappears.**
(TIF)

Figure S3 **Bundle-mediated stalk formation.**
(TIF)

Figure S4 **Additional bundle-mediated fusion simulations.**
(TIF)
