# Correction: Line-Tension Controlled Mechanism for Influenza Fusion

**DOI:** 10.1371/annotation/14cda8c1-a852-4f1f-b00c-bc45e48c8305

**Published:** 2013-10-15

**Authors:** Herre Jelger Risselada, Giovanni Marelli, Marc Fuhrmans, Yuliya G. Smirnova, Helmut Grubmüller, Siewert Jan Marrink, Marcus Müller

The captions of supporting information files Figures S1-S4 were erroneously omitted. The supporting information captions read:

Figure S1: Calculation of the line tension. The figure shows the X and Y-dimensions of an periodic (in Z-dimension) elongated stalk (A) and a lambda-shaped membrane (B) formed between two bilayers with broken periodicity in the X-dimension. The simulation box length in the Z-dimension is constant and pressure coupling is only present in the X,Y plane. The broken periodicity in the X-dimension is required to allow free membrane shape deformations and redistribution of material to ensure on overall tension-less conditions. As the resulting bilayer edges and the elongated stalk are within the same direction the line tension of the elongated stalk is given by: λ_stalk_ = λ_total_ - ("number of edges")×λ_edge_. Here, λ is derived from the box dimensions (L) and the pressure tensor (P) as: λ = L_XX_L_YY_ × (P_ZZ_ − 1/2(P_XX_ + P_YY_)). λ_edge_ is obtained by a separate simulation of the corresponding bilayer edge.

Figure S2: Remaining lipid splay connecting the two adjacent DOPC leaflets before the stalk completely disappears.

Figure S3: Spontaneous stalk formation between a POPE vesicle and POPE bilayer in the vicinity of the peptide pore. Shown is the onset of stalk formation (1.8 μs). The presence of the pore and surrounding peptides enhance local bilayer perturbations and lower inter-membrane repulsions by increasing the hydrophobicity (cyan colored) of the fusion site. Spontaneous bending of the target membrane allows the pore edge to come into close proximity with the vesicle. The cyan colored lipids are ’splayed’ lipids that are about to form ’lipid bridges’ between the adjacent leaflets. Sidechains of the peptides are colored yellow, backbone red.

Figure S4: Influenza mediated fusion between a small vesicle and bilayer. (A:I-III) Fusion reaction between a small POPC vesicle and DOPC membrane. The bundle recovers itself after fusion. (B) The stalk surrounds a formed hole. Some peptide’s are leaving the ’free’ rim of the hole. (C) Expanded HD observed in the fusion reaction with a POPE vesicle. Notice, the recovered bundle near the fusion site. (D) Despite the high curvature stress in the small POPE vesicle, the absence of the peptides/pore inhibits further progression of fusion between a DOPC membrane and POPE vesicle on the time scale of 8μs. Influenza fusion peptides are shown in red, interior solvent is shown in blue, PC head-groups in

honeydew, and PE head-groups in orange. 

