# Supplementary figures and images for: Line-Tension Controlled Mechanism for Influenza Fusion
Source: PLoS One. 2012 Jun 28;7(6):e38302. doi: 10.1371/journal.pone.0038302 (PMC3386277; doi:10.1371/journal.pone.0038302)

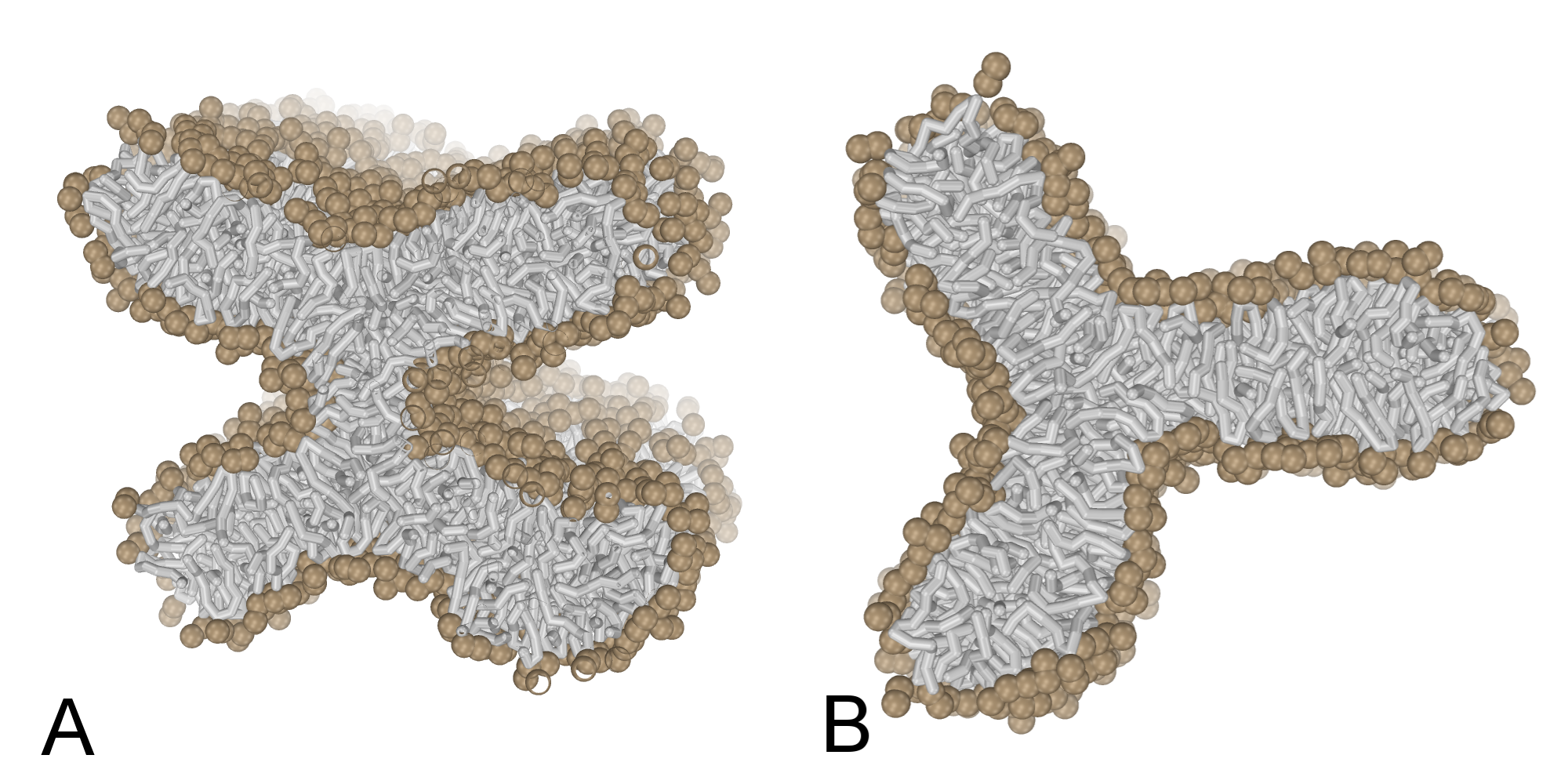

Supplement: Figure S1 — Calculation of the line-tension of the elongated stalk and HD. (TIF) [file pone.0038302.s001.tif]

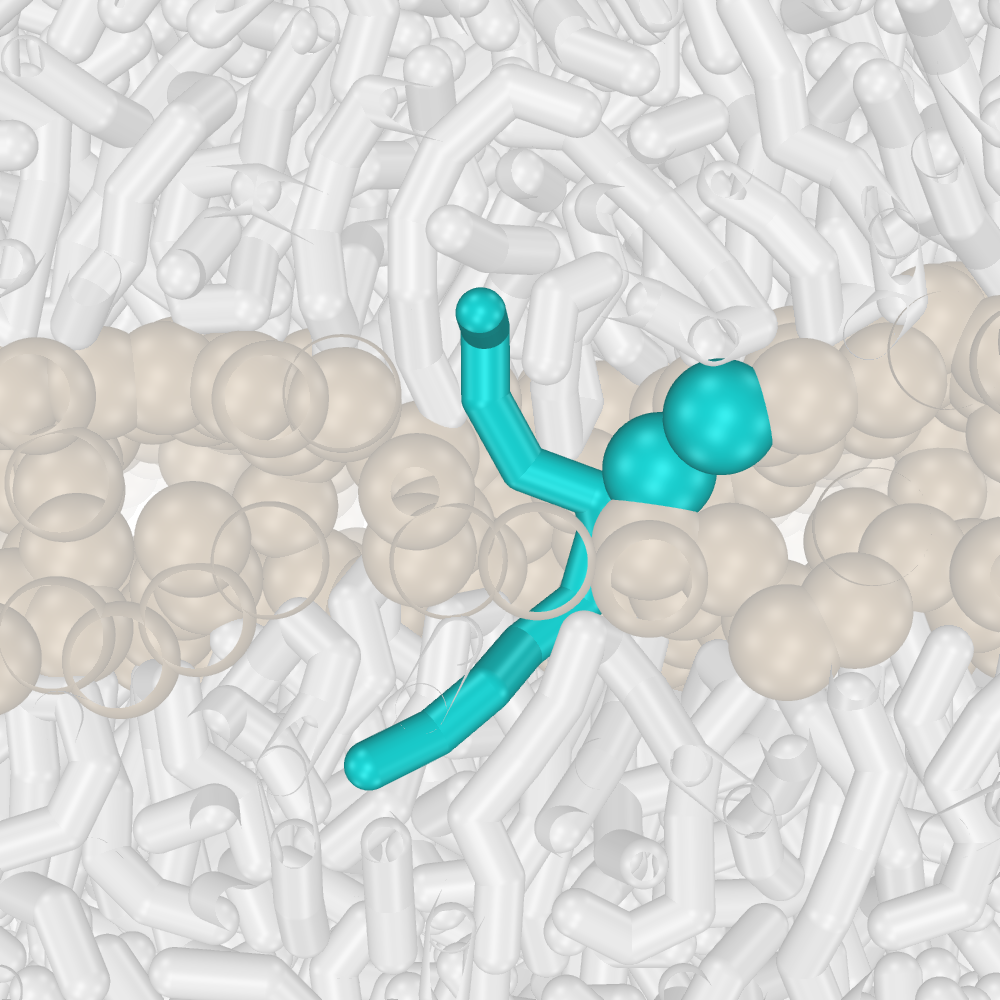

Supplement: Figure S2 — Splayed lipid intermediate observed before the stalk disappears. (TIF) [file pone.0038302.s002.tif]

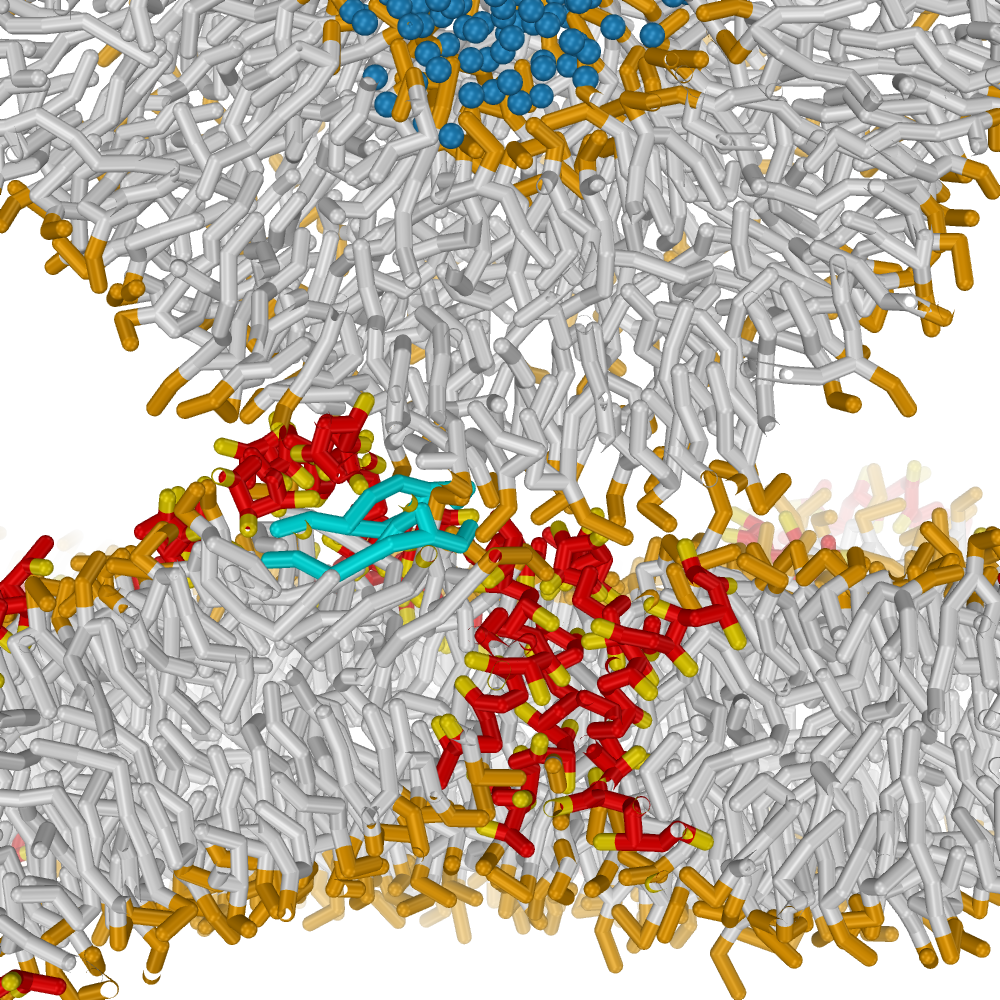

Supplement: Figure S3 — Bundle-mediated stalk formation. (TIF) [file pone.0038302.s003.tif]

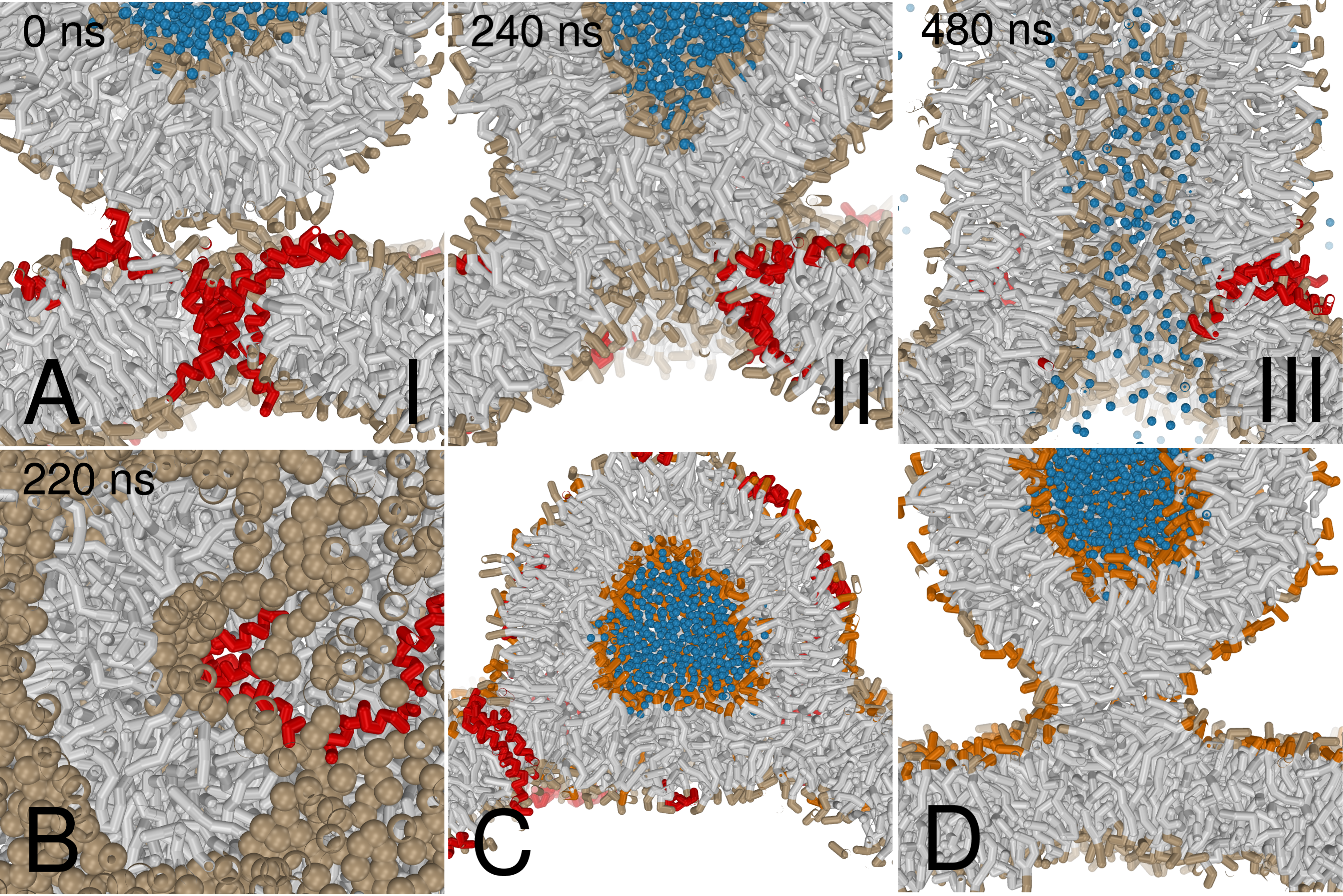

Supplement: Figure S4 — Additional bundle-mediated fusion simulations. (TIF) [file pone.0038302.s004.tif]
